# Genetic Determination of a Cryptic Species in the *Littoraria* Genus With Whole‐Genome Molecular Resolution

**DOI:** 10.1002/ece3.70715

**Published:** 2024-12-10

**Authors:** Jia‐Wei Xu, Jie Wang, Yun‐Wei Dong

**Affiliations:** ^1^ The Key Laboratory of Mariculture, Ministry of Education, Fisheries College Ocean University of China Qingdao People's Republic of China; ^2^ Function Laboratory for Marine Fisheries Science and Food Production Processes Pilot National Laboratory for Marine Science and Technology Qingdao People's Republic of China

**Keywords:** cryptic species, *Littoraria*, morphological differences, Northwest Pacific, whole‐genome molecular data

## Abstract

Recognizing cryptic species is crucial for understanding global biodiversity. The intertidal snail *Littoraria flammea* is potentially a cryptic species of *L. melanostoma* widely distributed in the Northwest Pacific. However, the evidence from traditional morphology and single genetic markers is inconsistent. Our study combined quantitative morphological and whole‐genome molecular data to clarify the phylogenetic relationship of three species ( *L. flammea* , *L.* aff. *melanostoma*, and *L. melanostoma* ). Three‐dimensional models of shells revealed significant differences in morphology between *L. flammea* and *L. melanostoma* . Neutral SNPs indicated that individuals of *L. flammea* and *L. melanostoma* were in different clusters. The ratio of interspecific *F*_ST_ to intraspecific *F*_ST_ between *L. flammea* and *L. melanostoma* (16) was much larger than the lowest ratio (2.31) in six published genera with cryptic species in gastropods. Non‐neutral SNPs disclosed divergence in functional genes related to reproduction and protein binding. The morphological and phylogenetic analyses corroborated the transitional status of *L.* aff. *melanostoma*. These results confirmed that the *L. flammea* snails north of the Yangtze River Estuary is a cryptic species of *L. melanostoma* , and allopatric speciation occurs in the *L. melanostoma* complex.

## Graphical abstract

Our study combined quantitative morphological and genome‐wide molecular data to elucidate the controversial phylogenetic relationships among three species of the Littoraria complex ( *L. flammea* , *L. melanostoma* , and L. aff. melanostoma) in the Northwest Pacific. Our findings confirm that the *L. flammea* snails north of the Yangtze River Estuary is a cryptic species of *L. melanostoma* , and dispersal and vicariance could influence allopatric speciation in the *L. melanostoma* complex.

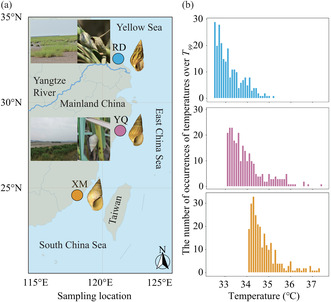

## 1 Introduction

Species are the fundamental units of biodiversity, yet even within well‐studied taxonomic groups, the fundamental task of species discovery often remains incomplete. While some difficulties arise from philosophical disagreements about species delimitation (De Queiroz 2007), more significant uncertainties stem from the misidentification of cryptic lineages that are phenotypically or ecologically similar to known species (Bickford et al. 2007; Struck et al. 2018). The cryptic species, which cannot readily be distinguished in morphology but are on different evolutionary trajectories, pose great challenges in assessing biodiversity. It is suggested that the magnitude of species diversity is underestimated in terrestrial vertebrate species (Moura and Jetz 2021) and marine invertebrate species (Appeltans et al. 2012; Chen 2021) due to the existence of these difficult‐to‐identify organisms. Identifying cryptic species helps to address various biological questions related to evolutionary parallelism, convergence, and conservation (Chan et al. 2022; Del Monte‐Luna et al. 2023; Delić et al. 2017; Fišer, Robinson, and Malard 2018). New threats posed by accelerated climate change and habitat loss increase the urgency of discovering and comprehensively documenting patterns of global biodiversity. (Bickford et al. 2007; Delić et al. 2017).

The discovery of more and more cryptic species in the Northwest Pacific (NWP) has attracted much attention in the study of marine biodiversity and mechanisms of speciation in this region (Siaden et al. 2019; Tang et al. 2010; Ujiié and Lipps 2009). Allopatric speciation is recognized as a predominant mechanism driving species formation in the NWP, resulting in a wealth of concealed biodiversity (Fernández‐álvarez et al. 2023; Muto and Kai 2023; Song et al. 2020). Complex historical events and present‐day physical environments in the NWP create unique conditions for allopatric speciation (Ekimova et al. 2021; Hyde and Vetter 2007; Shen, Chang, and Durand 2015). The physical conditions include seasonal ocean currents, hydrography, tidal cycles, different substrate types, and discharges from major rivers like the Yangtze River (Hu and Dong 2022; Ni et al. 2017; Williams et al. 2019). For example, Cheng and Sha (2017) unveiled two cryptic species within the *Oratosquilla oratoria* species complex, with one cryptic species (lineage N) in the cold‐water area within the temperate zone and another (lineage S) in a warm‐water region influenced by the Kuroshio Current. Their divergence was hypothesized to be a result of an isolation event in the Sea of Japan during the middle Pliocene. Their study also suggested that riverine outflow from the Yangtze River may have acted as a marine barrier to facilitate geographic segregation and allopatric diversification of the *O. oratoria* species complex. As increasing studies report cryptic species in this region, it is necessary to determine cryptic species and reassess biodiversity using newly developed technologies (Korshunova et al. 2019; Taylor et al. 2019; Trivedi et al. 2016).

The advent of sequencing technology provides an opportunity to revisit known species through multiple analytical perspectives and detect cryptic species (Bickford et al. 2007; Satam et al. 2023). Early phylogenetic analyses can reveal interspecific phylogenetic relationships and intraspecific population structures with genetic markers such as mitochondrial DNA (mtDNA), nuclear ribosomal DNA (nrDNA), plastid DNA (ptDNA), and low‐copy‐number genes (Lee and Wen 2001; Li et al. 2008; Moritz 1994; Stefanović et al. 2008). These different markers, however, are ill‐suited for identifying cryptic species, since they give rise to inconsistent results due to hybridization and incomplete lineage sorting (Osborne et al. 2016). To address the challenges of systematics and phylogenetics related to species boundaries and species‐level relationships, researchers have increasingly turned to the utilization of whole‐genome genetic markers (Barrow, Lemmon, and Lemmon 2018; Jones et al. 2016; Valencia et al. 2018; Young and Gillung 2020; Yu et al. 2018). The next‐generation sequencing (NGS) methods, which are known for their efficiency and sensitivity in providing genome‐wide markers, have found widespread application in biodiversity research and have proven instrumental in uncovering cryptic species (Elfekih et al. 2018; Herrera et al. 2022; Rubinoff et al. 2020). One of the NGS methods is the genotyping by sequencing (GBS) technology, which stands as a potent instrument for surmounting the limitations of traditional approaches when dealing with cryptic species and subtle genetic differentiations between species (Cronemberger et al. 2020; Viard, David, and Darling 2016). For instance, three cryptic species within the earthworm genus *Carpetania* (Oligochaeta, Hormogastridae) were determined by GBS (Marchán et al. 2020), facilitating their integration into ecological research and biodiversity conservation initiatives.

Snails of the genus *Littoraria* are widely distributed in the NWP. The morphological characteristics, phylogenetic relationships, and geographic distribution ranges of most *Littoraria* species have been described in detail (Reid 1986, 1999a, 1999b, 2001; Stuckey and Reid 2002). However, there are some controversies about cryptic species within this genus (Reid, Dyal, and Williams 2010). According to the morphological diagnostic characteristics, including the shell and reproductive anatomical features (e.g., the shape of the penis), the number of species has been estimated at 39, plus two subspecies (Reid 1986). Reid, Dyal, and Williams (2010) reconstructed the phylogeny of the genus *Littoraria* using molecular markers (28S, 12S, and COI). Their results showed that the phylogenetic relationships based on the molecular information were largely similar to those from traditional morphology, except that *L. flammea* might be a cryptic species of *L. melanostoma* (Reid, Dyal, and Williams 2010). The snail *L. flammea* was recognized as one extinct species (Carlton 1993; Carlton et al. 1999; Dulvy, Sadovy, and Reynolds 2003; Dulvy, Pinnegar, and Reynolds 2009; Monte Luna et al. 2007). Dong, Huang, and Reid (2015) reported the rediscovery of *L. flammea* in salt marshes near Shanghai in China. According to the shell morphology, they suggested that there might be three species in the NWP: one was *L. flammea* in Rudong, north of the Yangtze River, one was *L. melanostoma* in Xiamen, south of the Yangtze River, and one was *L*. aff. *melanostoma* between Xiamen and Rudong. Unfortunately, analysis of COI, 12S, and 28S gene sequences did not perfectly support the separation of these three *Littoraria* species.

In the present study, we combined quantitative morphological and high‐throughput sequencing (GBS) methods to understand whether the three species ( *L. flammea* , *L. melanostoma* , and *L*. aff. *melanostoma*) are cryptic species or different populations of a species. We expect that our integrated methods could efficiently distinguish cryptic species in the intertidal zone and that the geographic distribution of cryptic species in the NWP could be explained by historical events and/or present physical environments.

## 2 Materials and Methods

### 2.1 Sample Collection

**FIGURE 1:**
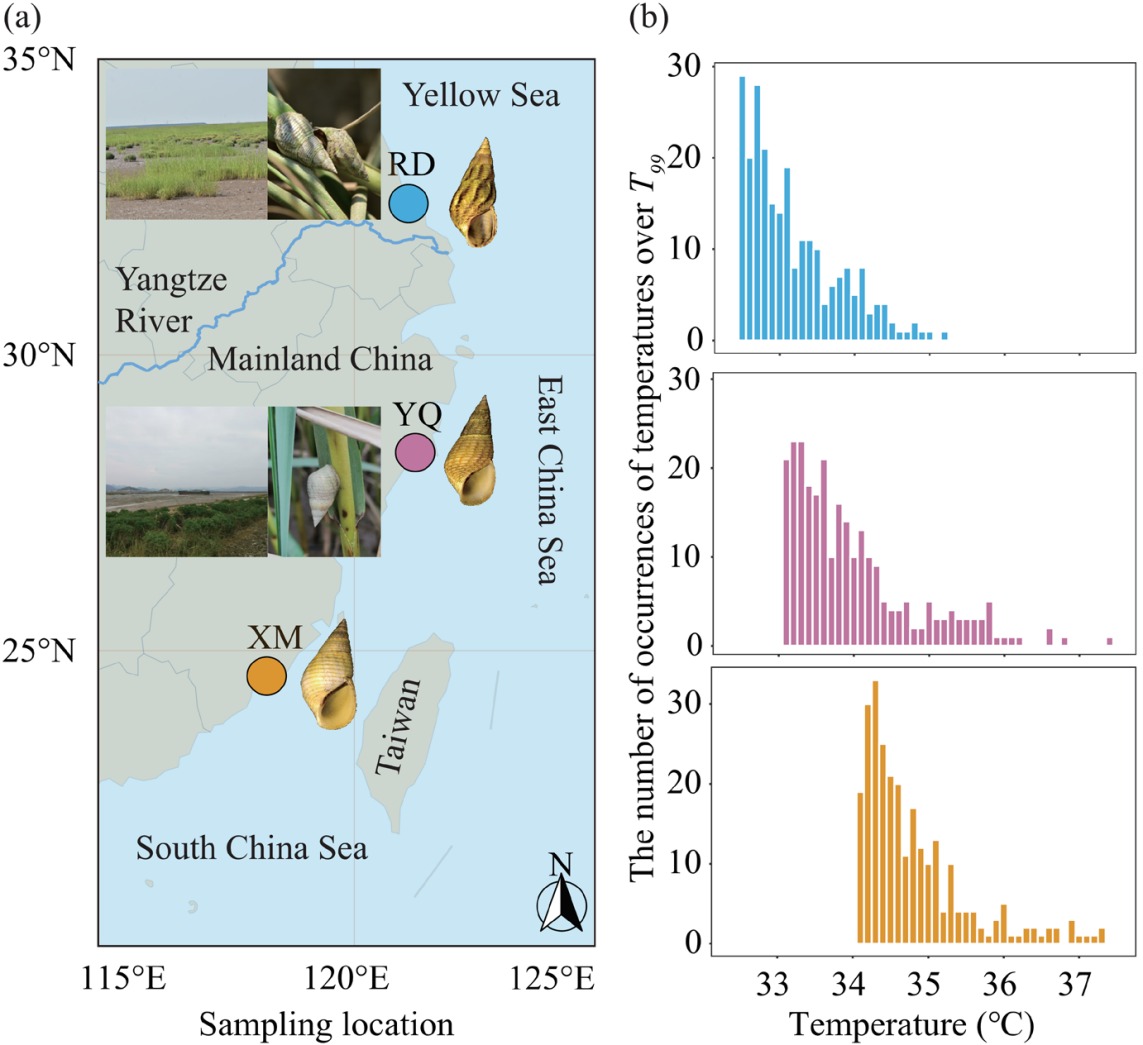
(a) The sampling locations of *Littoraria flammea*, *L*. aff. *Melanostoma*, and *L. melanostoma* in Rudong (RD), Yueqing (YQ), and Xiamen (XM), respectively. (b) The number of occurrences of each temperature exceeding the 99th percentile temperatures (*T*_99_) in summer (June–September) from 2011 to 2020 in each locality.

The present study collected *Littoraria flammea*, *L*. aff. *melanostoma*, and *L. melanostoma* specimens from Rudong (RD; 32.56°N, 121.06°E), Yueqing (YQ; 28.35°N, 121.19°E), and Xiamen (XM; 24.57°N, 118.29°E), respectively, along the coastline of China in January 2021 (Figure 1a). We noticed that each sampling location exclusively hosted a single species and species' habitat conditions were different (Figure 1a). Specifically, *L. flammea* primarily inhabited the leaves and stems of low smooth cordgrass in RD, while *L*. aff. *melanostoma* was found on the leaves and stems of tall smooth cordgrass and mangrove in YQ. The snail *L. melanostoma* in XM occurred on artificial coastal defense structures and mangroves.

### 2.2 Variation Analysis of Extreme Temperatures

There is a latitudinal cline in environmental thermal heterogeneity in the Northwest Pacific, which affects sensitivity of populations to high temperatures (Dong, Han, and Huang 2014), divergence of populations (Wang, Cheng, and Dong 2022), and even cryptic speciation (Cheng and Sha 2017). Therefore, it is necessary to consider temperature variations in studying the process of speciation in this region. Given that temperature extremes hold significance in unraveling the effect of temperatures on species distributions (Helmuth et al. 2002), we compared variations in extreme temperatures among sampling locations. We retrieved and analyzed hourly temperature data measured 2 m above the ground from the National Centers for Environmental Prediction's Climate Forecast System Reanalysis (CFSR). Then, the 99th percentile of temperatures (*T*_99_) for the summer months (June–September) spanning from 2011 to 2020 was calculated as the decade's highest temperature that the snails experienced, representing extreme thermal stress. The Kruskal–Wallis test was employed to ascertain the significance of variations in extreme temperatures across various sampling locations within the *T*_99_ dataset.

### 2.3 Comparison of Shell Morphology

Although the characteristics of shell morphology of *L. flammea* and *L. melanostoma* were well described by Reid (1986), there was a lack of a quantitative method to distinguish these species in shell form. Here, we constructed 3D models of snail shells based on a previously described method (Larsson et al. 2020) using the *ShellShaper* software (https://github.com/jslarsson/ShellShaper). This method focused on the outer shell of a 3D object by capturing and characterizing the shape, size, curvature, and other features of snail shells. It included parameters for height and width growth, which describe the shape of the shell, as well as aperture characteristics such as position (radial position in Larsson et al. 2020), size and shape (aperture extension in Larsson et al. 2020). We captured images of snail shells using a Nikon camera (D3500) equipped with a macro lens (AF‐S MICRO NIKKOR 105 mm) and then loaded the images into the software for further analysis. Specifically, we determined an initial point at the apex of the shell, and then the extreme points on the right and left edges of the most recent whorls (see Figure S1). Then, a one‐parameter family of egg‐like shapes (named “circlipses”) was used to depict variable aperture forms by smoothly combining a half‐circle with a half‐ellipse. Finally, these key points and curves were utilized to fit the shell morphology based on the Thin‐Plate Spline model for shape interpolation. We selected six key parameter values from the resulting models for the morphological comparison: the shell length (*L*), shell width (*g*_*w*_), shell height (*g*_*h*_), radial distance from the origin to the helix at the current aperture position (*r*_0_), vertical distance from the origin to the helix at the current aperture position (*h*_0_), and the circlipse size parameter (*a*_0_) (Figure S2). Since these parameters depend on both shell length and aperture size, the statistical differences observed between species could be driven by size variation alone. To account for this, we followed the approach of previous studies (Koch et al. 2021; Larsson et al. 2020; Raffini et al. 2023) and applied size corrections to the aperture position parameters (*r*_0__scaled = *r*_0_/*L*, *h*_0__scaled = *h*_0_/*L*), aperture size (*a*_0__scaled = *a*_0_/*L*), height growth (log_*g*_*h*_), and width growth (log_*g*_*w*_).

### 2.4 Genotyping by Sequencing (GBS) and Genome‐Wide SNP Calling

In the absence of a reference genome, genotyping by sequencing was used to obtain genome‐wide SNPs of study species. We prepared a pooling GBS library containing a total of 65 samples, including 24 individuals of *L. flammea* , and 15 individuals of *L*. aff. *melanostoma*, and 26 individuals of *L. melanostoma* . Our process began with the extraction of genomic DNA from muscle tissue using the E.Z.N.A. Tissue DNA Kit (Omega Bio‐Tek) following the manufacturer's instructions. Subsequently, restriction digestion was performed using EcoRI and DpnII enzymes (New England Biolabs). Different 12‐bp barcode sequencing adapters were ligated to digested DNA to distinguish different samples, and PCR was carried out to enrich each library. We then purified each library and selected fragments within the desired size range (100–500 bp). Finally, all libraries were pooled and sequenced using the Illumina NovaSeq 6000 sequencing system (Shanghai BIOZERON Co. Ltd.) with a 150‐bp paired‐end protocol. The sequencing results of the pooled libraries were split based on different adaptor pairs to obtain the raw data for each sample.

To obtain high‐quality clean reads, the software *fastp* v.0.22.0 (Chen et al. 2018) was used to remove the last 30 bp of raw reads. The clean reads from all three species specimens were used for de novo assembly with *Stacks* v.2.62 (Catchen et al. 2011). SNP calling was conducted using *Stacks* with default parameter settings except for *M* = 3 (number of additional allowed differences between stacks within individuals) and *n* = 3 (number of allowable differences between stacks among individuals).

### 2.5 Neutral SNPs and Phylogenetic Analysis

To obtain high‐quality neutral SNPs, we performed strict filtering based on previously described methods (Thorstensen, Baerwald, and Jeffries 2021; Wang, Cheng, and Dong 2022). Firstly, *VCFtools* v.0.1.16 (Danecek et al. 2011) was employed for filtering and main settings were as follows: biallelic SNPs; minor allele frequency (MAF) ≥ 0.05; loci with genotypes called in at least 80% of individuals; mean depth of coverage across all individuals ≥ 10; genotypes called with a quality score ≥ 20; Hardy–Weinberg Equilibrium (HWE) at *p* < 0.005. Furthermore, SNPs that had high linkage disequilibrium (LD) were pruned (*r* ^2^ = 0.2) using the *SNPRelate* v.1.20.1 (Zheng et al. 2012).

The obtained neutral SNPs were used to assess genetic diversities and phylogenetic relationships among three species. Private alleles (*N*), observed heterozygosity (*H*_*o*_), expected heterozygosity (*H*_*e*_), nucleotide diversity (*π*), inbreeding coefficient (*F*_IS_), and polymorphic loci were calculated using the “‐‐genepop” command within the *Stacks* subroutine *populations*. The phylogenetic tree was constructed using two distinct methods. An unweighted pair grouping method with arithmetic mean (UPGMA) in the *phangorn* v.2.11.1 (Schliep 2011) was used to generate dendrograms based on Hamming genetic distances. One thousand bootstrap iterations were run to assess the robustness of branching patterns. Additionally, a maximum likelihood tree was built based on the general time reversible model with a single rate per locus (GTR + CAT) using *FastTree* software (Price, Dehal, and Arkin 2009).

Two methods were employed to infer the overall pattern of genetic structure and individual ancestry. Firstly, we used a model‐based approach implemented in *ADMIXTURE* v.1.3.0 (Alexander, Novembre, and Lange 2009) to infer individual ancestries within a maximum likelihood (ML) framework. The *K* values ranged from 1 to 10, and ancestry coefficients (*Q*) were estimated with 10 repetitions per *K*. In addition, we performed a discriminant analysis using the principal component discriminant analysis (DAPC) function in the *adegenet* package v.2.0.2 (Jombart 2008). The dataset was transformed using PCA, and *K*‐means clustering with BIC was applied to determine the optimal number of clusters among *K* values ranging from 1 to 10. We assessed genetic differentiations between species by pairwise *F*_ST_ values using *genepop* v.1.2.2 (Rousset, Lopez, and Belkhir 2020). Given that *F*_ST_ values may be influenced by demographic factors such as small population size or limited connectivity between populations, we also calculated absolute population divergence (*d*_XY_) among the three species with *pixy* v.1.2.10.beta2 (Korunes and Samuk 2021). Lower connectivity or smaller population size are not expected to result in higher *d*_XY_ values, which are instead closely linked to the time since divergence (and may be reduced by gene flow occurring after divergence). To statistically test for admixture in *L*. aff. *melanostoma*, we utilized the *f3* statistics from the *Admixtools* package v.2.0 (Maier et al. 2023). The *f3* estimates if a given population is the result of an admixture between two other populations. Here, the *L. flammea* and *L. melanostoma* populations in the dataset were used as sources of admixture, while the *L*. aff. *melanostoma* population served as the target population.

### 2.6 Outlier SNPs and Annotations

To assess non‐neutral genome‐wide variations among species, we employed three statistical methods to identify putative outlier SNPs. Firstly, we identified outlier SNPs in terms of population structure by conducting PCA using *pcadapt* v.4.3.3 (Luu, Bazin, and Blum 2017). Based on the results of PCA, the two principal components (*K* = 2) captured most of the background genetic variation. The SNPs that significantly deviated from the neutral background structure (Bonferroni correction, adjusted *p* < 0.001) were identified as potential genetic differentiation loci. Secondly, we employed the *F*_ST_ sliding window approach to identify genomic regions under diverse selection. Weighted *F*_ST_ in 50,000‐bp windows (10,000‐bp window steps) was analyzed using the *weir‐fst‐pop* function of *VCFtools*. We selected locus windows whose weighted *F*_ST_ values were in the top 99th percentile for each paired‐population selection analysis and analyzed the *F*_ST_ of the SNPs within these windows in ranked order. We considered any SNP within these windows that lay above the *F*_ST_ threshold associated with each paired dataset as a putative outlier SNP. Thirdly, outlier SNPs were identified based on the Bayesian hierarchical model implemented in *BayPass* v.2.31 (Gautier 2015). The core model in *BayPass* was run four times with default settings of nval of 100,000, burnin of 50,000, npilot of 30, and pilotlength of 5000, and the results of the runs were averaged. Calibration of the XtX statistic was performed according to the manual by creating a pseudo‐observational dataset using the simulate.baypass function, and subsequently running it on the core model using the same settings, calculating thresholds of 1% and 99% to distinguish between neutral and outlier loci. SNPs with XtX statistics above the 99% threshold and below the 1% threshold (representing directional and balanced selection, respectively) were considered outliers. To reduce the impact of false positive outlier SNPs on subsequent analyses, this study only analyzed the SNPs in the intersection. Since there were no outlier SNPs in the intersection of all three methods, we proceeded with the analysis using only the outlier SNPs that appeared in the intersection of any two methods, according to the suggestions by previous studies (Vu et al. 2020; Wang et al. 2023).

The genes containing outlier SNPs were annotated with the Non‐Redundant (NR) database on the National Center for Biotechnology Information (NCBI) website using Blastx. Functional annotations of these genes were obtained and analyzed for gene ontology (GO) enrichment using the *Blast2GO* software (Conesa et al. 2005).

### 2.7 Collection of *F*_ST_ Data on Inter‐ and Intraspecies Genetic Distances in Cryptic Species

To assess whether a gastropod could be classified as a cryptic species based on neutral SNPs, we reevaluated inter‐ and intraspecific genetic distances from previous publications on cryptic species study. We used keywords such as “cryptic species,” “*F*_ST_,” “SNP,” and “Gastropods” to search in the Web of Science and Google Scholar. Since one piece of literature did not provide inter‐ and intraspecies genetic distances of cryptic species, we downloaded the raw data from NCBI and calculated the *F*_ST_ values using the methods mentioned above (see Sections 2.4 and 2.5).

## 3 Results

### 3.1 Latitudinal Variation in Thermal Environments

The Kruskal–Wallis test was employed to assess latitudinal variations in temperature extremes among the three sampling locations. The results revealed significant differences in extreme temperature (*T*_99_) during the summer over the past 10 years (2011–2020) for each pair of the three sampling locations (*p* < 0.05). The location RD exhibited significantly lower temperature extremes (*T*_99_ = 32.38°C) compared to the other two locations (YQ: *T*_99_ = 33.06°C; XM: *T*_99_ = 34.10°C) (Figure 1b). There were 32 instances (11.9% of the observations) and 103 instances (38.3%) of extreme heat events exceeding 34°C in RD and YQ, respectively, suggesting high variations in thermal environments among three locations.

### 3.2 Morphological Divergence

**FIGURE 2:**
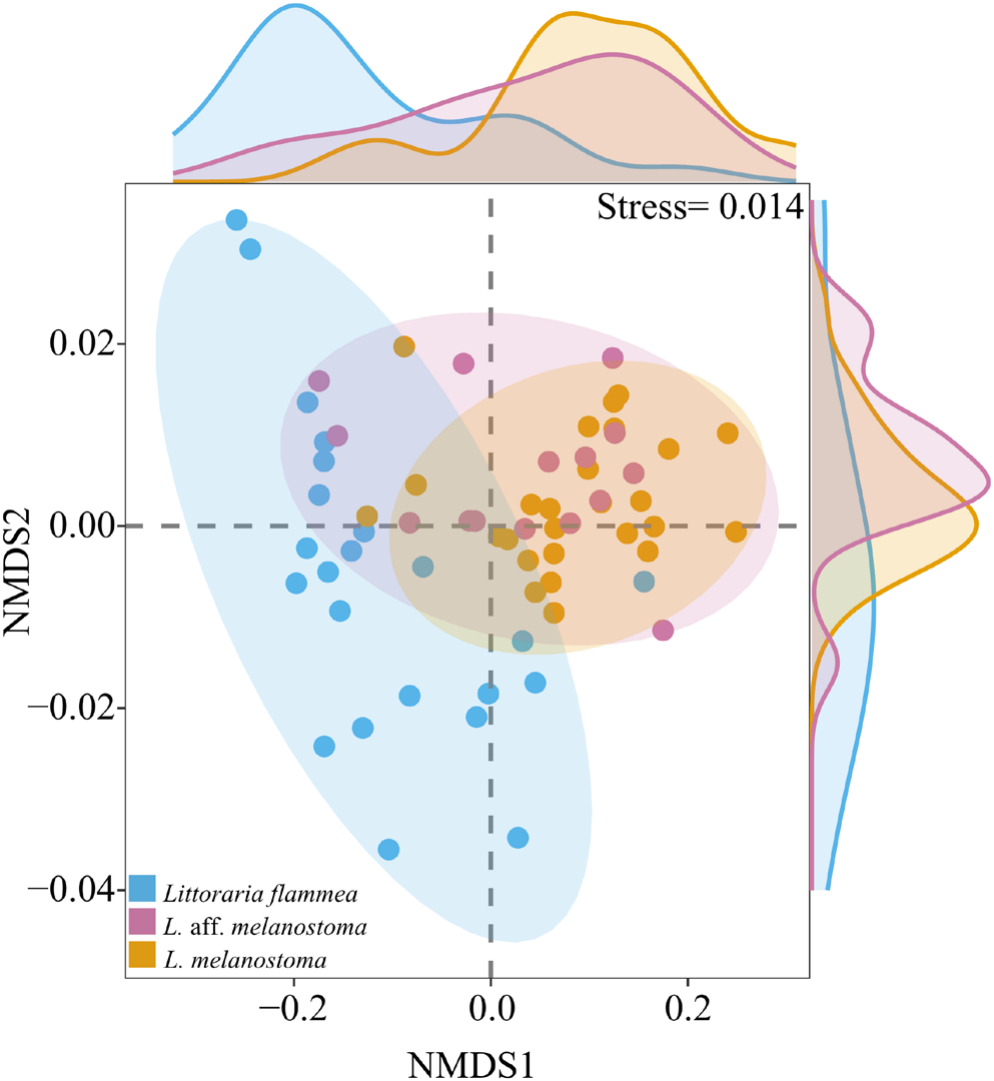
A 2D nonmetric multidimensional scaling (nMDS) scatter plot relating to shell morphology for three *Littoraria* species. Ellipses with different colors indicate 95% confidence ellipses for centroids of morphological parameters. The stress value, indicating the goodness of fit, is remarkably low at 0.014. The density profiles on the right and top of the figure highlight areas of maximum concentration of shell parameters along the different NMDS axes. Notably, shell parameters along the NMDS1 axis play a key role in distinguishing *L. flammea* from the other two snail species.

One‐way ANOVA analysis revealed significant differences in three of six shell morphology parameters among three species (Table 1). The shell length (*L*) and growth parameters (*log_g*_*h*_ and *log_g*_*w*_) in *L. flammea* were significantly smaller than those in the other two species (Table 1). No significant differences were observed between *L*. aff. *melanostoma* and *L. melanostoma* . There were no significant differences in the aperture position parameters (*r*_0__scaled and *z*_0__scaled) and aperture size (*a*_0__scaled) among the three species. The nonmetric multidimensional scaling (nMDS) revealed two distinct clusters with a stress value of 0.014: cluster 1 contained *L. flammea* , while cluster 2 included *L*. aff. *melanostoma* and *L. melanostoma* (Figure 2).

**TABLE 1:** Shell parameters (mean ± SD) of three *Littoraria* species.

Species	*L*	*r*_0__scaled	*h*_0__scaled	*a*_0__scaled	−log_*g*_*h*_	−log_*g*_*w*_
*Littoraria flammea*	17.15 ± 1.64^a^	0.117 ± 0.010^a^	0.837 ± 0.029^a^	0.181 ± 0.017^a^	1.150 ± 0.029^a^	1.231 ± 0.055^a^
*L*. aff. *melanostoma*	19.50 ± 1.86^b^	0.118 ± 0.010^a^	0.826 ± 0.019^a^	0.184 ± 0.011^a^	1.072 ± 0.025^b^	1.166 ± 0.040^b^
*L. melanostoma*	20.44 ± 1.69^b^	0.120 ± 0.013^a^	0.826 ± 0.016^a^	0.190 ± 0.013^a^	1.084 ± 0.026^b^	1.172 ± 0.029^b^

*Note:* ‘a’ and ‘b’ indicate statistically significant groupings. At the *p* < 0.05 level, the same letter is not significant, while different letters indicate significant differences.

### 3.3 Neutral SNPs and Phylogenetic Relationships of Three Species

A total of 7354 neutral SNP loci were retained in 65 individuals of three snail species. Genetic diversity analysis revealed that the expected heterozygosity (*H*_*e*_) and nucleotide diversity (*π*) were smaller in *L. flammea* (*H*_*e*_ = 0.149 ± 0.002, *π* = 0.153 ± 0.002), in comparison with *L*. aff. *melanostoma* (*H*_*e*_ = 0.183 ± 0.002, *π* = 0.191 ± 0.002) and *L. melanostoma* (*H*_*e*_ = 0.190 ± 0.002, *π* = 0.194 ± 0.002) (Table S1). The percentage of polymorphic loci in *L. flammea* (64.67%) was also lower than that in the other two species (*L*. aff. *melanostoma*, 77.17%; *L. melanostoma* , 83.77%). The snail *L*. aff. *melanostoma* had only one private allele, while *L. flammea* and *L. melanostoma* had 872 and 125 private alleles, respectively.

**FIGURE 3:**
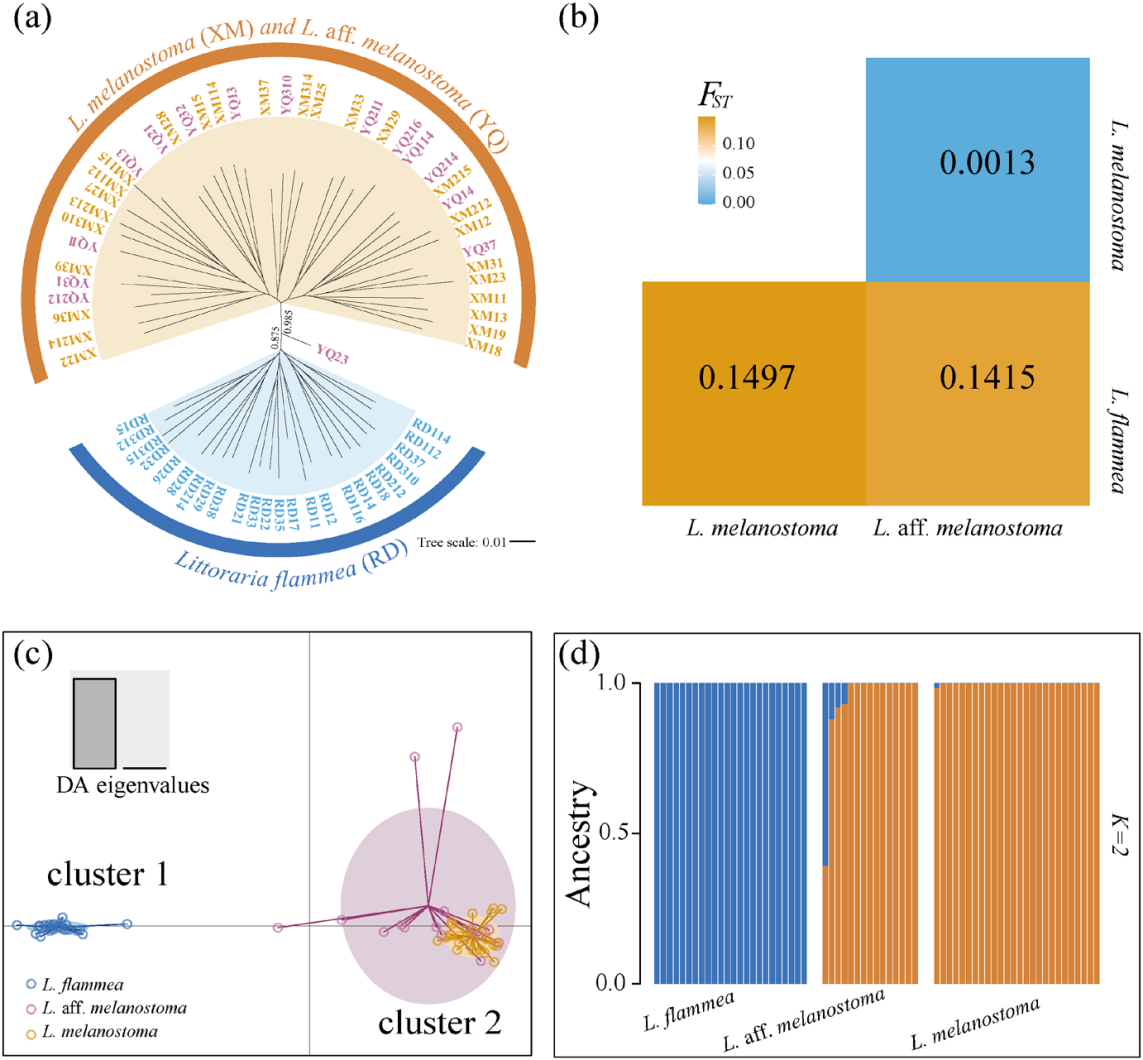
Phylogenetic relationships of three snail species. (a) The phylogenetic maximum likelihood (ML) tree depicts the relationships among three species: *L. flammea* (RD), *L*. aff. *melanostoma* (YQ), and *L. melanostoma* (XM). The support values on the branches are based on 1000 bootstrap replicates. (b) Pairwise values of *F*_ST_ based on neutral SNPs. (c) Discriminant analysis of principal components (DAPC) for the neutral SNPs. The axes are the first two linear discriminants or DA eigenvalues (901.920 and 3.274, respectively). Each point on the plot represents an individual snail. The analysis revealed two clusters: Cluster 1 consists of *L. flammea* individuals and cluster 2 includes *L*. aff. *melanostoma* and *L. melanostoma* individuals. (d) Bar graph of *ADMIXTURE* analysis. The bars represent the proportions of each individual's genetic ancestry from the two ancestral populations (i.e., blue and orange colors represent different ancestral populations).

There was a substantial phylogenetic signal among the three *Littoraria* species. The maximum likelihood phylogenetic tree (ML tree) (Figure 3a) and the UPGMA consensus tree (Figure S3) unveiled two distinct groups: the first group contained exclusively *L. flammea* individuals and the second one included *L*. aff. *melanostoma* and *L. melanostoma* individuals. The genetic differentiation analysis showed that pairwise *F*_ST_ values between *L. flammea* and *L. aff. melanostoma* and *L. melanostoma* were 0.1415 and 0.1495, respectively, while the value was only 0.0013 between *L*. aff. *melanostoma* and *L. melanostoma* (Figure 3b). The DAPC clustering analysis also identified two distinct clusters (Figure 3c). Cluster 1 comprised *L. flammea* individuals, and cluster 2 encompassed all individuals of *L*. aff. *melanostoma* and *L. melanostoma* . The *ADMIXTURE* estimated the admixture proportion for the samples, and the optimal number of subpopulations was *K* = 2 (Figure S4). It not only distinguished *L. flammea* from *L. melanostoma* but also elucidated the intermediate nature of *L*. aff. *melanostoma*, since some individuals of *L*. aff. *melanostoma* exhibited genetic admixture patterns (Figure 3d). The *f3* statistic results further supported the possible hybridization event in the *L*. aff. *melanostoma* population (Table S2). Specifically, when *L. flammea* and *L. melanostoma* populations were source species, the *f3* value was −0.00155 ± 0.00871 (Mean ± SE), suggesting that hybridization between the *L. flammea* and *L. melanostoma* populations contributed to the genetic composition of *L*. aff. *melanostoma*. Negative *f3* values with *Z*‐values below −2 indicated weak but notable evidence of gene flow from these two source populations into *L*. aff. *melanostoma*. In contrast, other *f3* tests involving combinations of the same populations yielded positive results (0.0628 ± 0.000743 and 0.00222 ± 0.000242) and *Z*‐values far from the significance threshold, suggesting an absence of significant admixture signals in these cases.

### 3.4 Outlier SNPs

**FIGURE 4:**
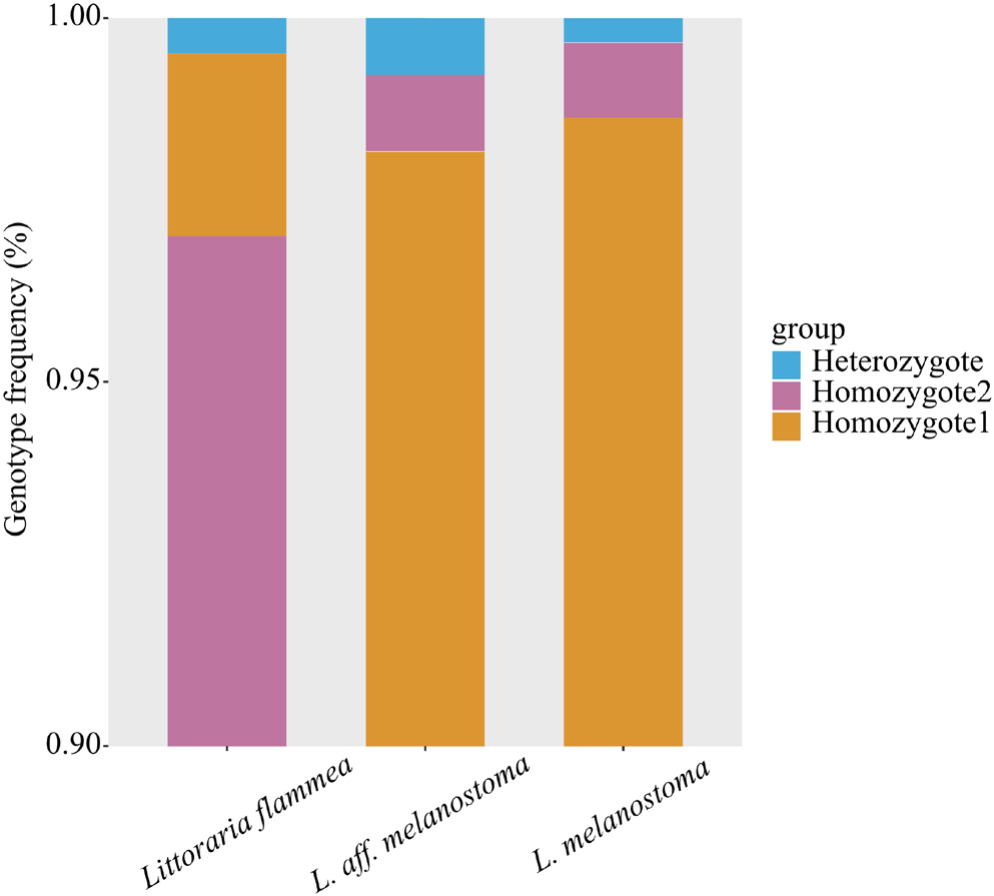
The percentage of genotypes among 4898 outlier SNPs under diversifying selection in three *Littoraria* species. Heterozygote is defined by the presence of one reference allele and one alternative allele, while homozygote encompasses either two reference alleles or two alternative alleles.

There were 4898 outlier SNPs detected by *F*_ST_, PCA, and *BayPass* methods (Figure S5). A total of 477 outlier SNPs (*F*_ST_ and PCA: 44, *F*_ST_ and *BayPass*: 9, PCA and *BayPass*: 424) were detected that appeared in any two detection methods (Figure S5). Among them, 57 SNPs were located on 34 genes with good annotation (Table S3). Most genotypes of *L. flammea* are Homozygote2 (97.0%), while the other two species predominantly consist of Homozygote1 ( *L. melanostoma* = 98.6%; *L*. aff. *melanostoma* = 98.2%) (Figure 4).

**FIGURE 5:**
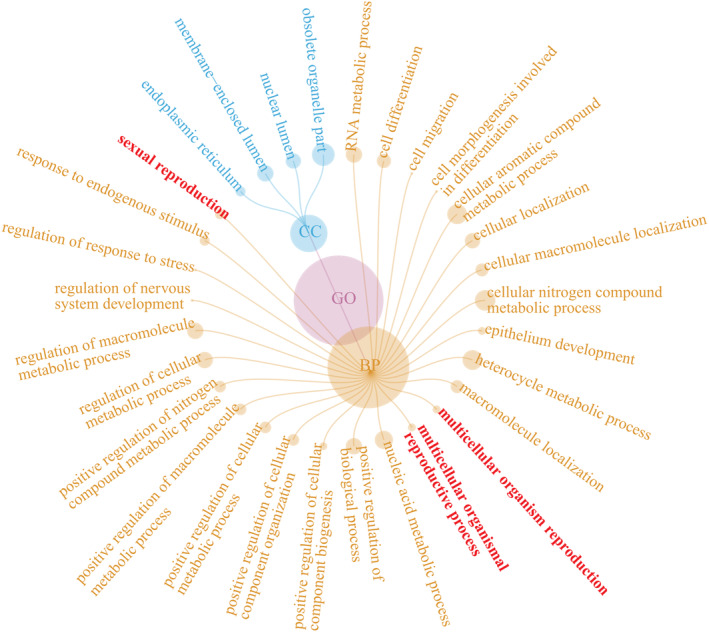
Top 30 Gene Ontology (GO) terms annotated for 34 genes containing outlier SNPs in three *Littoraria* species.

The 34 genes containing outlier SNPs were enriched in 118 GO terms. Among the top 30 significant GO terms, 26 terms were related to biological processes (BP), and four terms to cellular components (CC) (Figure 5). The BP terms included the sexual reproduction, the multicellular organismal reproductive process, and the multicellular organism reproduction. The CC terms contained obsolete organelle part, membrane‐enclosed lumen, nuclear lumen, and endoplasmic reticulum.

### 3.5 Genetic Distances Between Cryptic Species of Gastropods

After searching for published literature, we got intraspecific and interspecific *F*_ST_ values for six genera with cryptic species in gastropods (Table 2). The results showed that the ratio of interspecific *F*_ST_ (e*F*_ST_) to intraspecific *F*_ST_ (a*F*_ST_) varied from 2.31 to 461. For the *Littoraria* genus in the present study, the e*F*_ST_/a*F*_ST_ ratio between *L. flammea* and *L*. aff. *melanostoma*, as well as *L. melanostoma* , were 16.71 and 115.38, respectively.

**TABLE 2:** Inter‐ and intraspecies *F*_ST_ statistics in the study of cryptic species in gastropods.

Genus	Interspecific *F*_ST_ (e*F*_ST_)	Intraspecific *F*_ST_ (a*F*_ST_)	Ratio (e*F*_ST_/a*F*_ST_)	Reference
*Alviniconcha*	0.842–0.922	0.0020–0.0220	38.27–461	Castel et al. 2022
*Ancylus*	0.551–0.804^a^	0.0261–0.0712^a^	7.73–30.79	Weiss, Weigand, and Leese 2022
*Buccinum*	0.572–0.619	0.0590–0.0980	5.84–10.49	Goodall et al. 2021
*Coralliophila*	0.063–0.235	0.0020–0.0240	2.63–117.50	Simmonds 2016
*Limacina*	0.293–0.410	0.0321–0.1270	2.31–12.77	Choo et al. 2023
*Physella*	0.270–0.440	0.0100–0.1000	2.70–44.00	Stanford et al. 2023
*Littoraria*	0.142–0.150	0.0013–0.0085	16.71–115.38	Present study

^a^ The *F*_ST_ values calculated using our pipeline with the raw data from the article.

For the *Littoraria* genus, the absolute population divergence *d*_*XY*_ between *L. flammea* and *L. melanostoma* (0.380) and between *L. flammea* and *L*. aff. *melanostoma* (0.370) was about twice that of between *L. melanostoma* and *L*. aff. *melanostoma* (0.187). Among the six genera with cryptic species collected in this study, only genus *Alviniconcha* had reported *d*_*XY*_ values. Their results showed *d*_*XY*_ values of three potential cryptic species (*A. kojimai*/*A. boucheti*, 0.031; *A. kojimai*/*A. strummeri*, 0.108; *A. boucheti*/*A. strummeri*, 0.031) based on ddRAD‐seq data.

## 4 Discussion

Identifying cryptic species is crucial for comprehending global biodiversity, especially in accurately predicting biodiversity loss due to climate change. By combining quantitative morphological and genome‐wide molecular data, we suggest that the snails in Rudong north of the Yangtze River estuary are the species *Littoraria flammea* (Philippi 1847–1850), representing a cryptic species, instead of a population of the widely distributed Indo‐Pacific species *L. melanostoma* . Complex historical events and present physical environments in the NWP could play important roles during the cryptic speciation in *Littoraria*. We propose that our integrated methods might be an effective way to distinguish cryptic species in intertidal gastropods.

Quantitative morphological analysis of the shell helps species identification in marine gastropods. Previously, Reid (1986, 2001) distinguished *L. flammea* along the coastline of the East China Sea and Yellow Sea from *L. melanostoma* found in southern China (Fujian and Taiwan) to India based on the description of morphological features such as shell length, aperture shape, and the number of spiral ribs. Here, we found significant differences in growth parameters and shell length between *L. flammea* and *L. melanostoma* , which verified the classification by Reid (1986, 2001). We noticed that all shell parameters of *L*. aff. *melanostoma* were similar to those of *L. melanostoma* , but it was still significantly different from *L. flammea* in terms of growth parameters and shell length. These results supported the hypothesis that *L*. aff. *melanostoma* in Yueqing might be a transitional status between *L. flammea* in Rudong and *L. melanostoma* in Xiamen (Dong, Huang, and Reid 2015). This quantitative method in shell morphology has been used in identifying different ecotypes of *Littorina saxatilis* adapted to wave action and crab predation on intertidal rocky shores (Hearn et al. 2022; Koch et al. 2021, 2022). Therefore, we suggest that this quantitative morphological analysis lies in its ability to provide objective and measurable data, facilitating a more rigorous and reproducible approach to species identification.

High‐throughput SNPs are useful in identifying cryptic species in gastropods. Previous studies (Dong, Huang, and Reid 2015; Reid, Dyal, and Williams 2010) failed to reveal the phylogenetic relationship between *L. flammea* and *L. melanostoma* by using single genetic markers. Our phylogenetic results based on neutral SNPs showed that *L. flammea* and *L. melanostoma* individuals belonged to different clusters. Population structure analyses also revealed high genetic divergence (*F*_ST_) and absolute population divergence (*d*_*XY*_) between *L. flammea* and *L. melanostoma* . Although high *F*_ST_ between them might be caused by reduced contemporary connectivity or low population sizes, high *d*_*XY*_ between them supported their deep divergence in history. The interspecific *F*_ST_ (e*F*_ST_) between the two *Littoraria* species was 0.1497, which was 16.71 times higher than the intraspecific *F*_ST_ (a*F*_ST_) in each species. To assess whether this difference is sufficient to distinguish cryptic species, we compared our results with the levels of e*F*_ST_ and a*F*_ST_ in six gastropod genera with cryptic species. We found a genetic difference between cryptic species was at least 2.31 times larger than that within species in all six genera. Given that the e*F*_ST_/a*F*_ST_ ratio was over 16 and high *d*_*XY*_, we suggest that the snails found in Rudong were *L. flammea* , not a divergent population, but a cryptic species of *L. melanostoma* in Xiamen. High‐throughput SNP genotyping offers unparalleled genomic resolution and allows for the detection of subtle genetic variations that may be imperceptible using traditional genetic markers (such as COI), thereby facilitating the discrimination of closely related species (Rovelli et al. 2019). Our *ADMIXTURE* result showed that some individuals in Yueqing exhibited genetically admixed characters, partially confirming that *L*. aff. *melanostoma* is a transitional status between *L. flammea* and *L. melanostoma* . The *f3* statistics (negative *f3* with *Z*‐values below −2) also supported that *L*. aff. *melanostoma* resulted from admixture between two other populations. The intermediate phenotypic and genotypic traits of *L*. aff. *melanostoma* might be not due to geographic isolation and the high geographic similarity between *L. flammea* and *L. melanostoma* , but rather to the existence of gene flow between the two species. Similar patterns have also been observed in some genera. For example, Zhang et al. (2019) suggested that there were transitional species between *Phylloscopus affinis* and *P. occisinensis* in their contact zone since seven individuals exhibited genetically mixed features between these two sister species.

Beyond species identification, high‐throughput SNPs allow for the detection of genomic signatures associated with local adaptation and speciation. In the present study, we annotated some genes with outlier SNPs. These genes were involved in multiple biological processes, such as multicellular organismal reproductive process and sexual reproduction, suggesting that the snails from three locations might diverge in reproduction. Such differences may represent ecological traits that have been differentiated throughout their evolution (Hendry, Nosil, and Rieseberg 2007; West‐Eberhard 2005). In addition, partial genes with outlier SNPs (e.g., *HSPA5*, *PKC*) were involved in molecular functions such as protein binding. Such genes are temperature‐sensitive genes and play crucial roles in response to heat stress (Mishra and Grover 2016; Mosser et al. 1997). For example, the *HSPA5* gene encodes the binding immunoglobulin protein, which plays a critical role in the endoplasmic reticulum heat stress response (Dong, Du, and Huang 2022; Santamaría et al. 2019). We suggest that the occurrence of outlier SNPs in these functional genes might reflect environmental selection and/or local adaptation since there were obvious variations in extreme temperature among three sampling locations. Extreme temperatures bring a strong selective pressure, so the difference in extreme temperatures among regions would cause the variation of some gene loci and further species evolution (Buckley and Huey 2016; Tan et al. 2023). Our study also reveals differences in the types of outlier SNP purities (two reference alleles vs. two alternative alleles) across the three species, potentially implying the existence of different selection pressures. Unfortunately, the present study cannot determine whether different heterozygosity of specific loci under divergence selection would affect species' local adaptation and evolution. Since the difference in heterozygote frequency of functional genes potentially alters adaptation potential (Mcgaughran, Laver, and Fraser 2021), it is necessary to study genotyping in future studies.

Multiple factors control population divergence and speciation of intertidal species in the NWP. The geographic distributions of *L. flammea* and *L. melanostoma* indicate that geographic distance, the Yangtze River Estuary, temperature variation, and historical events play important roles in the cryptic speciation of *Littoraria*. The snail *L. flammea* was only found in Rudong north of the Yangtze River estuary, while the northern limit of *L. melanostoma* was in Ningde (Chen and Song 1988), 300 km north of Xiamen. It has been suggested that speciation in tropical, planktotrophic littorinids (such as *Echinolittorina*) was typically an allopatric process, requiring at least 1200 km of geographic isolation (Williams and Reid 2004). Likewise, species differentiation in *Littoraria* also exhibited allogamy, with six out of seven known sister species pairs retaining some geographic signal (Reid, Dyal, and Williams 2010). Since most sister species pairs in the *Littoraria* genus had partial geographic overlap and the geographic signal largely disappeared at deeper levels within the phylogeny, speciation in this genus may not have required such long geographic isolation as 1200 km (Reid, Dyal, and Williams 2010). Therefore, a distance of ~900 km between Ningde and Rudong might be sufficient for the cryptic speciation of *Littoraria*. Secondly, the Yangtze River discharge acts as a barrier to gene flow and might accelerate species differentiation. The Yangtze River estuary has been proven an important barrier in the formation of biogeographic and phylogeographic structure of intertidal species in the NWP (Dong et al. 2012; Ni et al. 2017; Wang, Tsang, and Dong 2015; Yu et al. 2014). Yu et al. (2014) identified three *Nipponacmea* species, which were once recognized as one species in China, and they suggested that present‐day physical conditions, including the Yangtze River discharge, played important roles in their speciation in this region. Temperature variation could also be a crucial physical factor leading to phenotypic and genotypic divergence in the Indo‐West Pacific region. A recent study found that differences in extremely high temperatures and their frequency between different locations in this region are important factors driving physiological and genotypic divergence in an intertidal snail *Nerita yoldii* that is gradually expanding into higher latitudes (Wang, Cheng, and Dong 2022). It has also shown that differences in high‐temperature environments are an important driving force behind adaptive genetic differentiation between cryptic species within certain genera, such as in the *Echinolittorina* genus in the Indo‐West Pacific region (Williams and Reid 2004) and the *Crepidula* genus in the Northeast shores of the United States (Wuitchik et al. 2024). In this study, although we lack direct evidence of the physiological or genetic impacts of high temperatures for the studied species, we observed differences in extreme temperatures of the habitats between *L. flammea* and *L. melanostoma* , as well as variations in the genotypic frequency of some temperature‐sensitive functional genes. We suggest that temperature may also be an important factor driving the emergence of the cryptic speciation of *Littoraria*. Additionally, historical events might have profound impacts on the genetic structure and evolutionary processes of cryptic species in the NWP. Areas and configurations of the marginal seas in the NWP have dramatically changed during the Pleistocene glacial–interglacial cycles (Imbrie et al. 1993). The larvae of coastal species could disperse northward from the South China Sea (SCS) to the East China Sea (ECS) at an interglacial stage. However, when the sea level fell about 120–140 m at a glacial stage, the ECS and the SCS were separated by a land bridge that connected the mainland and Taiwan (Kimura 2000). The land bridge plays an important role in the present‐day distribution of genetic variation (Ni et al. 2014) and allopatric differentiation (Zhang 2020) of coastal species. Therefore, we suggest that dispersal and vicariance influence population genetic structure and allopatric speciation in the *Littoraria* genus in the NWP. That is, *L. melanostoma* widely distributed in the SCS in history expanded its range into the ECS by larval diffusion during interglacial periods, while the land bridge limited gene flow during glacial periods and caused genetic divergence within this species. After the Last Glacial Maximum, the individuals surviving along the Yellow Sea coast of China were isolated from the ones in the ECS by the Yangtze River discharge. In the evolutionary process, temperature variations among different marginal seas acted as thermal selection and enhanced divergence.

There is still considerable debate regarding the methods for defining cryptic species and whether the term “cryptic species” should continue to be used (Korshunova and Martynov, 2024). Based on the current research on cryptic species and the findings of our study, we propose several key aspects for future research in this field. First, the importance of morphological and anatomical analysis should not be overlooked. Developing more precise quantitative methods and utilizing advanced imaging techniques (such as computed tomography [CT] and magnetic resonance imaging [MRI]) to identify distinguishing tissue structures may help to effectively differentiate discrepancies that are currently difficult to resolve. Second, with the decreasing cost of whole‐genome sequencing and the widespread adoption of related methods, further structural variation analysis between species, based on chromosomal‐level genomes (not limited to SNPs), should be conducted. This approach will aid in clarifying the relationships between closely related species and the processes of speciation. Third, the development of a more systematic evaluation framework and methods (e.g., phyloperiodic approach by Korshunova and Martynov, 2024) to analyze difficult‐to‐differentiate species from multiple perspectives is necessary. The widespread adoption of these systematic research methods may eventually provide a more precise definition of when and how the term “cryptic species” should be applied.

In conclusion, we proposed that the integrated quantitative morphological and high‐throughput sequencing methods could be useful in identifying different *Littoraria* species (cryptic species). Both dispersal and vicariance could influence allopatric speciation in the *Littoraria* genus in the Northwest Pacific. The rediscovery and confirmation of *L. flammea* would enrich speciation and biodiversity studies in the NWP region, as well as contribute to the meaningful implementation of conservation measures for *L. flammea*.

## Author Contributions

**Jia‐Wei Xu:** data curation (lead), formal analysis (lead), investigation (lead), methodology (lead), software (lead), visualization (equal), writing – original draft (equal). **Jie Wang:** conceptualization (equal), formal analysis (supporting), methodology (supporting), supervision (equal), visualization (supporting), writing – review and editing (equal). **Yun‐Wei Dong:** conceptualization (equal), funding acquisition (lead), project administration (lead), supervision (equal), writing – review and editing (equal).

## Conflicts of Interest

The authors declare no conflicts of interest.

## Supporting information


Figures S1–S5.

Tables S1–S3.

## Data Availability

Raw sequence reads used in this article have been deposited in the National Center for Biotechnology Information Sequence Read Archive under BioProject PRJNA1050744.
