# Supplementary material for: Genetic Determination of a Cryptic Species in the Littoraria Genus With Whole‐Genome Molecular Resolution
Source: Ecol Evol. 2024 Dec 10;14(12):e70715. doi: 10.1002/ece3.70715 (PMC11631568; doi:10.1002/ece3.70715)
Supplement: Supplementary file 1 — Figures S1–S5. [file ECE3-14-e70715-s001.pdf]

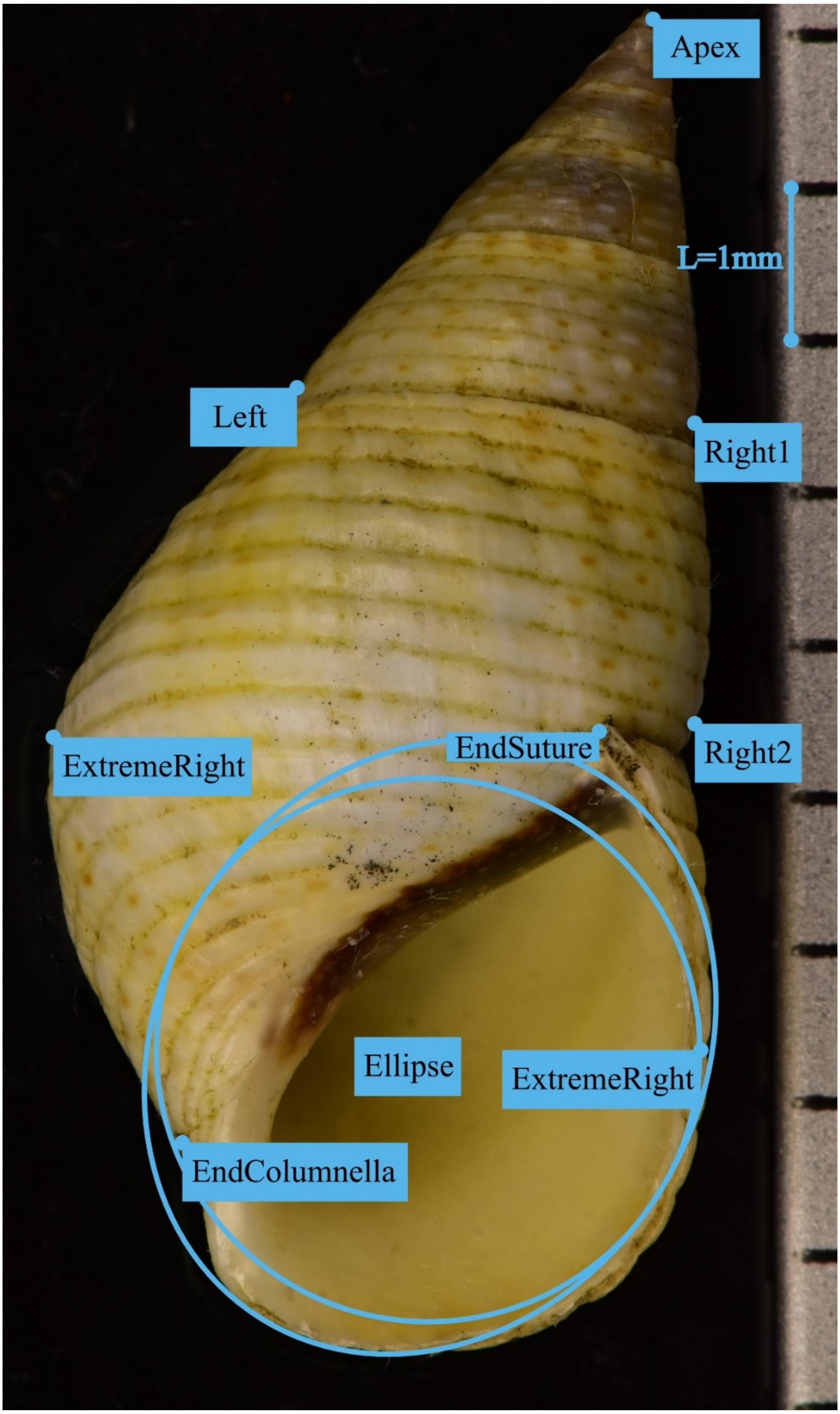

Figure S1. Input image and interactive positioning of objects.

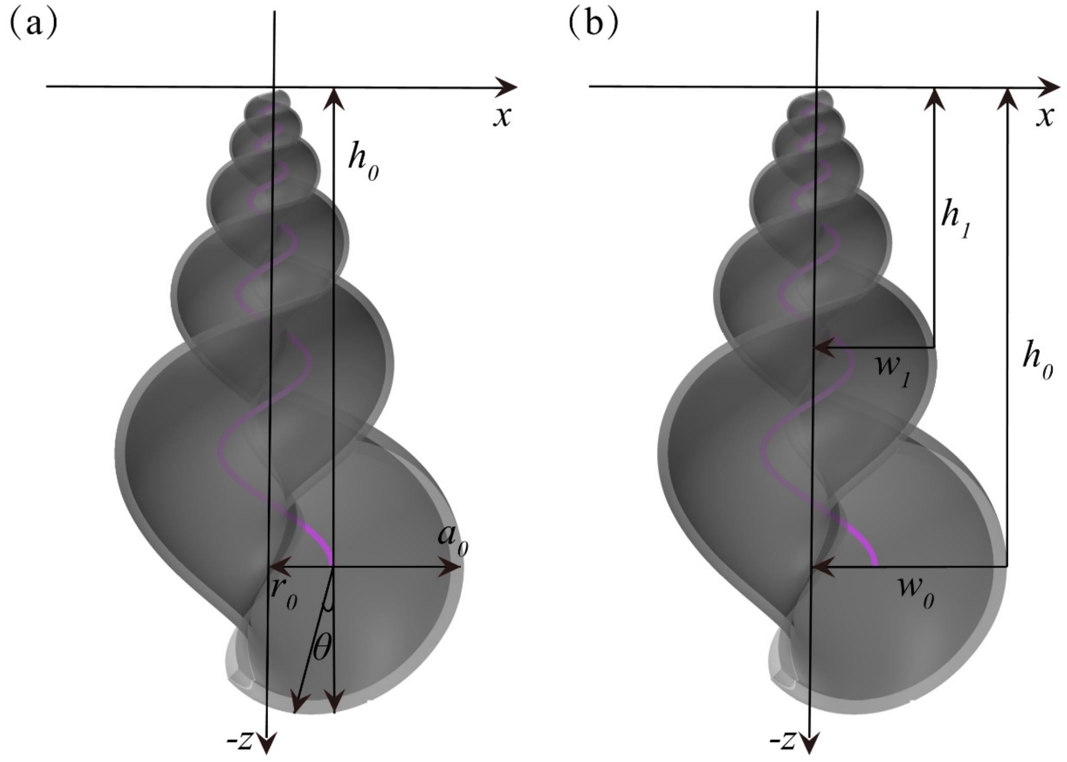

Figure S2. The semi-transparent shell model generated by *ShellShaper*. The internal spiral is highlighted in purple. The growth parameters can be calculated as $g_w = \ln(w_0/w_1)/(2\pi)$ and $g_h = \ln(h_0/h_1)/(2\pi)$. The circliptic aperture is rotated by θ relative to the internal spiral's normal plane defined by its normal and binormal, here rescaled to reach the aperture.

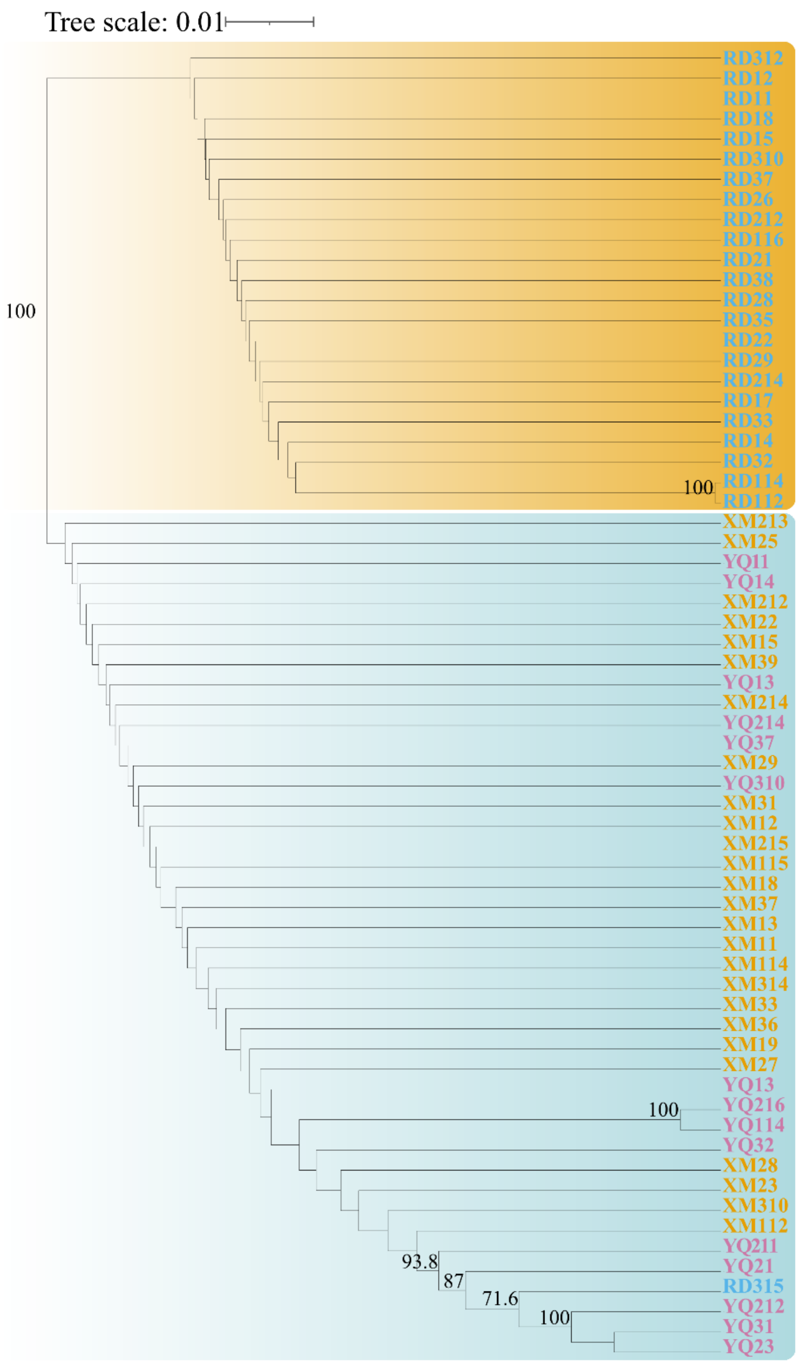

Figure S3. UPGMA consensus tree after 1,000 Bootstrap repetitions illustrating the relationships among individuals of the three snail species. Bootstrap values on nodes exceed 70%. In the figure, RD stands for *L. flammea*, YQ for *L.* aff. *melanostoma*, and XM for *L. melanostoma*.

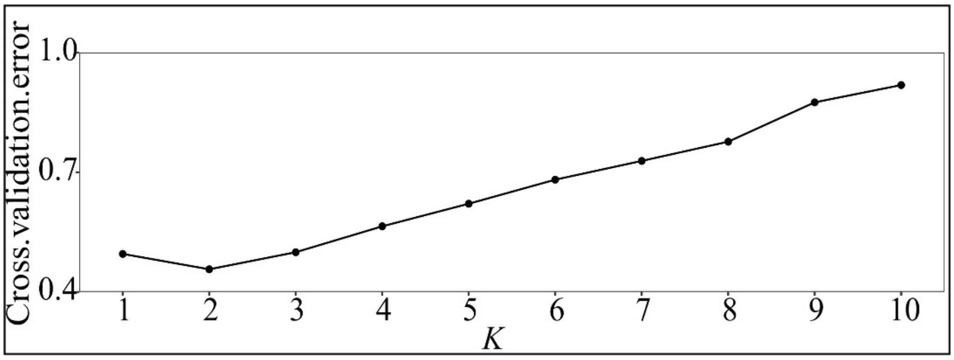

Figure S4. The cross-validation error rates for *K* = 1 to *K* = 10. The lowest error rate is observed when *K*=2, indicating that this value provides the best fit to the data and most accurately reflects the underlying genetic structure of the snail populations.

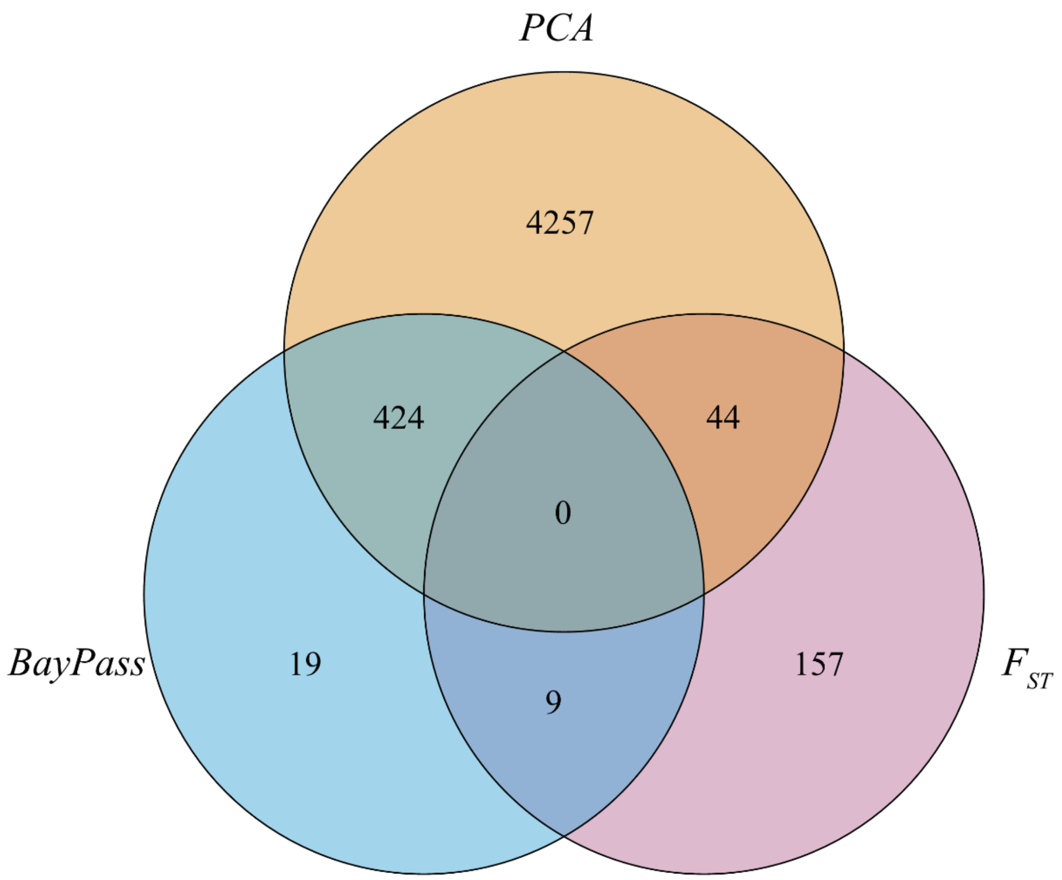

Figure S5. The Venn diagram shows the number of outlier SNPs screened by *PCA*, F_{ST}, and *BayPass*.
