# Supplementary material for: Genetic Determination of a Cryptic Species in the Littoraria Genus With Whole‐Genome Molecular Resolution
Source: Ecol Evol. 2024 Dec 10;14(12):e70715. doi: 10.1002/ece3.70715 (PMC11631568; doi:10.1002/ece3.70715)
Supplement: Supplementary file 2 — Tables S1–S3. [file ECE3-14-e70715-s002.pdf]

Table S1. Summary of diversity indices based on 7,354 neutral SNPs from GBS data for three snail species

**Specie**	**Private alleles (N)**	**Observed heterozygosity (H_o)**	**Expected heterozygosity (H_e)**	**Nucleotide diversity (π)**	**Inbreeding coefficient (F_{is})**	**Polymorphic loci (%)**
L. flammea	872	0.122±0.002	0.149±0.002	0.153±0.002	0.126±0.023	64.67
L. aff. *melanostoma*	1	0.164±0.002	0.183±0.002	0.191±0.002	0.128±0.022	77.17
L. melanostoma	125	0.163±0.002	0.190±0.002	0.194±0.002	0.089±0.020	83.77

Table S2. *f3* statistics testing for hybridization between populations.

**Source 1**	**Source 2**	**Target**	***f3***	**SE**	**Z**
L. flammea	*L. melanostoma*	*L.* aff. *melanostoma*	-0.00155	0.00871	-2.09
L. flammea	*L. flammea*	*L.* aff. *melanostoma*	0.0628	0.000743	0.779
L. melanostoma	*L. melanostoma*	*L.* aff. *melanostoma*	0.00222	0.000242	0.0569

Note: SE represents the standard error. Z represents the Z-score value.

Table S3. Overview of genes with good annotation and associated outlier SNPs

Outlier SNP ID	Annotation	REF	ALT	RD Ho_1	RD He	RD Ho_2	XM Ho_1	XM He	XM Ho_2	YQ Ho_1	YQ He	YQ Ho_2
contig103879_344	TRUB2	A	G	0	0	22	21	0	0	10	0	0
contig103879_367	TRUB2	A	T	0	0	22	21	0	0	10	0	0
contig103879_64	TRUB2	T	C	0	0	22	23	0	0	13	0	0
contig106299_110	Cytoplasmic Linker Associated Protein 1	G	A	0	0	23	26	0	0	12	0	0
contig106299_128	Cytoplasmic Linker Associated Protein 1	C	A	0	0	23	26	0	0	12	0	0
contig113869_173	B cell affinity maturation	A	T	0	0	17	24	0	0	14	0	0
contig11746_118	HSPA5	T	C	0	0	24	25	0	0	13	0	0
contig11746_97	HSPA5	A	G	0	0	24	26	0	0	13	0	0
contig14669_147	Mediator Complex Subunit 15	A	G	0	0	17	25	0	0	14	0	0
contig172959_195	metalloendopeptidase activity	A	T	0	0	21	24	0	0	12	0	0
contig172959_202	metalloendopeptidase activity	T	A	0	0	21	23	0	0	13	0	0
contig18442_40	MMS19	C	T	0	0	20	26	0	0	12	0	0
contig190918_8	COPI Coat Complex Subunit Gamma 1	C	T	0	0	19	23	0	0	9	0	2
contig19413_270	Zinc finger protein	C	A	0	0	24	25	0	0	14	0	0
contig29465_186	MTRR	G	A	0	0	24	26	0	0	14	0	0
contig29465_208	MTRR	A	G	0	0	24	26	0	0	14	0	0
contig32824_8	PDCD6IP	A	G	0	0	20	26	0	0	11	0	0
contig38019_158	UTP23	A	G	0	0	23	26	0	0	13	0	0
contig38019_21	UTP23	A	C	0	0	23	26	0	0	13	0	0
contig38871_315	Protein Kinase C Delta	A	G	0	0	22	22	0	0	11	0	0
contig38871_388	Protein Kinase C Delta	T	C	0	0	22	22	0	0	11	0	0
contig39991_101	Rab GTPase binding	A	C	0	0	19	22	0	0	13	0	1
contig39991_165	Rab GTPase binding	G	A	0	0	19	22	0	0	13	0	1
contig39991_167	Rab GTPase binding	A	T	0	0	19	22	0	0	13	0	1
contig39991_198	Rab GTPase binding	T	G	0	0	19	19	1	0	6	4	4

contig39991_216	Rab GTPase binding	C	T	1	6	12	22	0	0	13	0	0
contig40268_147	ERCC Excision Repair 5, Endonuclease	T	C	0	0	20	23	0	0	11	0	0
contig40619_277	GPI-anchor transamidase activity	A	G	0	0	20	25	0	0	11	0	0
contig41477_76	EF-Hand Calcium Binding Domain 5	C	T	0	0	23	23	0	0	15	0	0
contig44907_130	Solute Carrier Family 25 Member 12	C	A	0	0	21	25	0	0	9	0	0
contig44907_158	Solute Carrier Family 25 Member 12	C	A	0	0	21	25	0	0	9	0	0
contig46054_129	Protein of unknown function (DUF3253)	G	A	0	0	22	25	0	0	14	0	0
contig46054_86	Protein of unknown function (DUF3253)	T	C	0	0	23	25	0	0	14	0	0
contig47042_59	ECSIT Signaling Integrator	T	C	0	0	23	25	0	0	12	0	0
contig47042_86	ECSIT Signaling Integrator	C	T	0	0	23	25	0	0	12	0	0
contig59015_53	Zinc finger, C2H2 type	T	C	0	0	23	26	0	0	11	0	0
contig67912_133	Oxysterol Binding Protein Like 8	G	C	0	0	21	22	0	0	11	0	0
contig74496_110	PKD1L1	G	A	0	0	20	24	0	0	13	0	0
contig74496_152	PKD1L1	G	A	0	0	23	24	0	0	13	0	0
contig74496_22	PKD1L1	G	A	0	0	23	24	0	0	13	0	0
contig74496_46	PKD1L1	G	A	0	0	23	24	0	0	13	0	0
contig74496_71	PKD1L1	G	A	0	0	23	24	0	0	13	0	0
contig76519_46	Transmembrane Protein 245	G	A	0	0	22	25	0	0	10	0	0
contig7783_160	RNA secondary structure unwinding	C	A	0	0	21	25	0	0	13	0	0
contig78627_5	fasciculation of sensory neuron axon	C	T	0	0	21	24	0	0	14	0	0
contig82241_356	Mediator Complex Subunit 15	C	T	0	0	24	26	0	0	15	0	0
contig82241_89	Mediator Complex Subunit 15	A	G	0	0	23	26	0	0	15	0	0
contig86224_61	Pantothenate Kinase 4	C	T	0	0	22	26	0	0	11	0	0
contig86729_17	von Willebrand factor D and EGF	C	T	19	2	2	21	0	0	9	0	0
contig9017_109	Source PGD	C	T	0	0	23	25	1	0	9	0	0
contig91863_182	Serine threonine-protein kinase tousled-like	T	A	0	0	20	25	0	0	10	0	0
contig919763_111	Beta-lactamase	G	A	0	0	14	26	0	0	13	0	0
contig94066_34	atypical protein kinase C	T	C	0	0	23	24	0	0	7	0	0
contig94066_59	atypical protein kinase C	G	A	0	0	23	24	0	0	7	0	0
contig94770_124	DDB1 and CUL4 associated factor 4	A	T	0	0	23	24	0	0	12	0	0

contig94770_34	DDB1 and CUL4 associated factor 4	T	C	0	0	23	24	0	0	12	0	0
contig95735_234	G-protein coupled receptor activity	G	A	0	0	22	21	0	0	12	0	0

Note: Ho represents the Homozygote. He represents the Heterozygote.
